# Working-Memory, Alpha-Theta Oscillations and Musical Training in Older Age: Research Perspectives for Speech-on-speech Perception

**DOI:** 10.3389/fnagi.2022.806439

**Published:** 2022-05-11

**Authors:** Ryan Gray, Anastasios Sarampalis, Deniz Başkent, Eleanor E. Harding

**Affiliations:** ^1^Department of Experimental Psychology, University of Groningen, Groningen, Netherlands; ^2^Research School of Behavioural and Cognitive Neurosciences, University of Groningen, Groningen, Netherlands; ^3^Department of Psychology, Centre for Applied Behavioural Sciences, School of Social Sciences, Heriot-Watt University, Edinburgh, United Kingdom; ^4^Department of Otorhinolaryngology, University of Groningen, University Medical Center Groningen, Groningen, Netherlands

**Keywords:** older adults, alpha, theta, working memory, musical training, speech-on-speech perception

## Abstract

During the normal course of aging, perception of speech-on-speech or “cocktail party” speech and use of working memory (WM) abilities change. Musical training, which is a complex activity that integrates multiple sensory modalities and higher-order cognitive functions, reportedly benefits both WM performance and speech-on-speech perception in older adults. This mini-review explores the relationship between musical training, WM and speech-on-speech perception in older age (> 65 years) through the lens of the Ease of Language Understanding (ELU) model. Linking neural-oscillation literature associating speech-on-speech perception and WM with alpha-theta oscillatory activity, we propose that two stages of speech-on-speech processing in the ELU are underpinned by WM-related alpha-theta oscillatory activity, and that effects of musical training on speech-on-speech perception may be reflected in these frequency bands among older adults.

## Introduction: The Ease of Language Understanding Model and Working Memory

Under ideal listening conditions, speech perception is considered automatic (Johnson and Ralston, 1994). With increasing complexity, such as in background noise, it is an active cognitive process (Heald and Nusbaum, 2014; see Mattys et al., 2012), and one’s ability to parse information relies on combined sensory and cognitive capacities (van Knijff et al., 2018). When the background masker is non-speech, such as a steady noise, the speech acoustic cues are limited due to direct obliteration from the masker. In such “energetic masking” (Brungart, 2001), speech perception relies on the construction of the speech meaning from the audible temporal and spectral sections of the target speech, using linguistic and semantic constraints, but otherwise no further interference comes from the masker. In contrast, in speech-on-speech perception — a special case of “informational masking” (Pollack, 1975)— where the masker can be a single-talker, multitalker, or babble (gibberish), further interference can occur due to the overlapping temporal and spectral properties of the target and masking speech, and linguistic and semantic content of the masker speech.

During speech-on-speech perception, spectral and temporal components of the speech streams can be used to segregate target and masker streams to better understand a target speaker. For example, increased spatial separation (Viswanathan et al., 2016) and speaker voice differences (Darwin et al., 2003; Başkent and Gaudrain, 2016) seem to improve performance. In segregating target and masker components, the listener also needs to actively inhibit interference from the masker (Tun et al., 2002). Hence, higher-order cognitive functions like selective attention (Oberfeld and Klöckner-Nowotny, 2016) and working memory (WM; Sörqvist and Rönnberg, 2012) are also engaged when speech is attended in the presence of competing speech.

The Ease of Language Understanding (ELU) model (Rönnberg et al., 2013, 2019, 2021) highlights the importance of sensory-cognitive integration in speech-on-speech perception. An initial sensory module that Rapidly, Automatically, and Multimodally Binds Phonological information (RAMBPHO) allows for easy lexical access and understanding of speech. Presbycusis (age-related hearing loss) or interference from background speech can cause a mismatch in phonological binding of input and the representations in long-term memory. The phonological mismatch can result in increased reliance on WM to process degraded speech through an interplay with semantic long-term memory and episodic long-term memory (ELTM) in which linguistic inferences are formed based on prior knowledge.

Working memory is central to goal-directed behaviors where information must be stored and manipulated (Chai et al., 2018), and contains a general executive attention component (Chung et al., 2018) linked to inhibitory control (Getzmann et al., 2018; Tiego et al., 2018). The model’s conception of WM also assumes a central pool of cognitive resources that may be allocated to either storage or processing of information. Consequently, increased processing demands lead to reduced storage capacity and vice versa. The ELU emphasizes the integral role of WM during speech perception, especially in the presence of interfering sounds, and posits two distinct functional processes that determine successful comprehension: pre-diction and post-diction. The pre-diction aspect of the ELU suggests that WM is automatically and implicitly involved in the top-down modulation of perceptual processing in terms of priming the cognitive system *via* resource, or attentional allocation and the gating of sensory input. This in turn results in easier RAMBPHO and quicker understanding. Conversely, when a speech-signal is degraded, the post-diction component of WM works to integrate limited perceptual information with accessible representations from semantic long-term memory and ELTM in a more effortful pursuit of understanding (Rönnberg et al., 2021).

Working memory is associated with alpha (8–12 Hz) and theta (4–8 Hz) oscillatory activity (Yurgil et al., 2020) and appears to be underpinned by a fronto-parietal network that incorporates cortical and subcortical regions (Eriksson et al., 2015). In general, alpha-activity underpinned by the prefrontal cortex (PFC) is associated with the gating of sensory information during WM updating (Manza et al., 2014; Misselhorn et al., 2019), and the functional inhibition of activity in brain regions not involved with stimulus processing (Klimesch et al., 2007). Theta-activity is thought to represent interactions between the PFC, posterior regions and hippocampus (Strunk et al., 2017) throughout short-term storage, manipulation and retrieval of information (Hsieh and Ranganath, 2014; Roux and Uhlhaas, 2014). Interestingly, functional differences in WM alpha-theta oscillations reported in the literature seem to coincide with components of the ELU’s explicit processing loop —pre-diction and post-diction— that are triggered by phonological mismatch.

We suggest that alpha-activity may govern pre-diction *via* increased listening effort, in which the active inhibition of distractor speech gates relevant information, allowing for more successful binding during RAMBPHO. During speech processing, increases in alpha power are linked to heightened listening effort, particularly in adverse conditions where the auditory signal is degraded (Obleser et al., 2012; Dimitrijevic et al., 2019; Paul et al., 2021). This alpha-activity is related to the gating of lexical integration and selective inhibition of task-irrelevant noise (Strauß et al., 2014a,b), which likely becomes more challenging in the presence of background speech.

Meanwhile, when the speech signal is increasingly degraded, enhanced theta-activity represents the manipulation of limited sensory information to successfully trigger and retrieve appropriate lexical and semantic representations in long term memory stores, which are then integrated to form an understanding. Furthermore, fronto-parietal theta-activity has been implicated in lexical access and retrieval (Meyer et al., 2015; Marko et al., 2019) specifically from ELTM (Roberts et al., 2018; Nyhus et al., 2019), as well as being involved in resolving lexical ambiguity (Strauß et al., 2014a).

**FIGURE 1:**
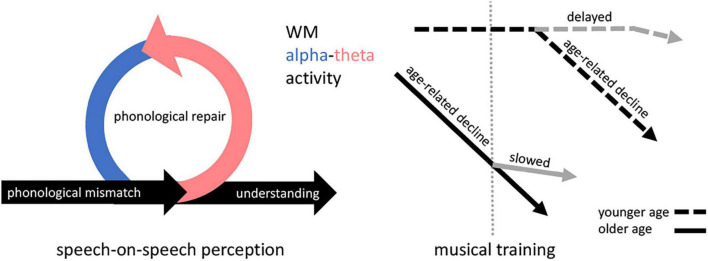
Hypothesized framework. Left, During speech-on-speech perception, the Ease of Language Understanding model (ELU; Rönnberg et al., 2019) describes how mismatch in phonological binding can occur which in turn triggers an explicit processing loop to repair the mismatch. The repair loop is governed by WM, consisting of the subcomponents pre-diction (blue) and post-diction (pink). Post-diction explicitly reevaluates lexical context, and pre-diction informs the (re-)binding of phonological information from the target speech signal in part by inhibiting distractor speech. Here, we suggest that the pre-diction and post-diction subcomponents of the loop are underpinned by alpha- and theta oscillatory activity, respectively. Right, We hypothesize further that any age-related decline in WM-related alpha-theta activity can be delayed if musical training occurred in younger age, or slowed by musical training in older age. In turn, musical training may preserve speech-on-speech perception longer into older age because preserved alpha-theta activity ensures proper repair of mismatched phonological binding. WM = working memory.

In sum, we propose that the dissociable roles of alpha and theta activity, apparent in WM subprocesses and highlighted during speech processing, directly underpin the pre-diction and post-diction components illustrated in the ELU (Figure 1), respectively. This pertains especially to speech-on-speech perception, which requires one to process a degraded speech signal and inhibit background speech. While this physiological underpinning seems intuitive and has in part been alluded to in previous ELU publications (Rönnberg et al., 2019), we will show in the coming sections how this phenomenon may be relevant for aging research. Namely, reduced WM-alpha-theta activity that supports pre-diction and post-diction processes after phonological mismatch may explain poorer speech-on-speech perception in older adults. Musical training is suggested in younger adult literature to be associated with increased alpha-theta activity and in older adult literature to enhance working memory. Thus (a background with) musical training in older adults may preserve alpha-theta oscillatory activity associated with working memory longer into older age (i.e., slow the decline). Preserved alpha-theta activity may accordingly preserve the ELU explicit-loop process that can repair phonological mismatch during speech-on-speech perception.

## Speech on Speech Perception and WM in Older Age

Aging affects sensory and cognitive mechanisms and can make speech-on-speech perception more challenging (Goossens et al., 2017). As the population continues to age and life expectancy becomes longer, addressing these challenges becomes even more relevant. Often these changes occur simultaneously, making it difficult to parse what limitations come from changes in sensory or cognitive mechanisms. On the sensory side, the incidence of hearing loss increases with advanced age (Cunningham and Tucci, 2017). In the population older than 65 years, fewer than 50% have normal hearing, which lowers to 10% in those older than 80 (Homans et al., 2017). Since speech-on-speech perception relies in part on parsing target and masker streams based on the spectrotemporal acoustic cues, e.g., pitch contours, common onset and offset, common modulations, voicing, and similar (Bregman, 1990), presbycusis could have a negative effect by potentially altering these cues. However, even when hearing loss is minimized, speech-on-speech perception has been shown to be difficult in middle-aged (Başkent et al., 2014) and older individuals (Zobel et al., 2019).

On the cognitive side, age-related changes have been observed as decline in specific functions such as speed of processing, WM, long and short-term memory (Salthouse, 1996; Deary et al., 2009; Murman, 2015; Pliatsikas et al., 2019), but not in crystallized functions such as verbal knowledge (Park and Gutchess, 2002), which likely contribute to older individuals compensating for other changes when understanding degraded speech (Saija et al., 2014; Başkent et al., 2016). Specifically related to WM, poorer speech perception in older adults has been linked to age-related changes in WM, and particularly poorer attentional control compared to younger adults (Goossens et al., 2017). Likewise, lower WM and inhibitory control performance for normal hearing older adults is associated with more profound difficulties during speech-on-speech (Vermeire et al., 2019), and auditory working memory scores have been observed as the strongest predictor of performance (Anderson et al., 2013). Thus from the perspective of the ELU, one contribution to impoverished speech-on-speech understanding in older adults could be reduced functionality in the WM-governed explicit processing loop consisting of pre-diction and post-diction that occurs after phonological mismatch (RAMBPHO).

Here we suggest a physiological framework for the ELU such that pre-diction and post-diction are underpinned by alpha and theta-oscillatory activity, respectively. This framework is consistent with neurophysiological accounts of WM decline in older adults as well. Working memory decline in older adults is marked by reductions in myelination in the PFC (Schulze et al., 2011), which in turn is associated with WM-related oscillatory activity in alpha- and theta bands (e.g., Strunk et al., 2017; Misselhorn et al., 2019). Accordingly, in older adults, observed decline in WM positively correlates with alpha-theta activity, and is predicted by parahippocampal, striatal, and superior longitudinal fasciculus white matter integrity (Steiger et al., 2019; Kumral et al., 2020). Moreover, where age-related WM decline is observed, associated modulations of alpha-theta activity could explain difficulties in speech-on-speech perception (see Strauß et al., 2014a,b). Slower resting-state alpha and lower alpha-theta power are reported in high WM-load tasks (Rondina et al., 2019), and alpha-power reduction accompanies increased susceptibility to distractions (ElShafei et al., 2020) such as speech maskers (Tun et al., 2002), which increases the likelihood of phonological mismatch during pre-diction. Older adults reputedly sometimes bind information from target and distractor streams, resulting in poorer recall (Strunk et al., 2017), possibly due to deficits in WM maintenance (Jarjat et al., 2019) and ELTM retrieval (Korkki et al., 2020) that are reflected by changes in frontal theta-activity (Tóth et al., 2014).

Taken together, we suggest that within the framework of the ELU, older adults who show signs of WM decline may have a reduced capacity to gate information and inhibit distractor speech due to reductions in alpha-activity, leading to poorer pre-diction. Additionally, declines in WM and subsequent theta-activity result in a lessened ability to maintain and manipulate a degraded speech signal during lexical retrieval, leading to poorer post-diction.

## Musical Training and WM in Older Age

Musical training is a complex activity that integrates multiple sensory modalities and higher-order cognitive functions (Olszewska and Marchewka, 2021). Musical training studies cannot provide definitive evidence as to whether cognitive benefits are exclusively attained through lifelong musical training, or whether they can be achieved through training once already in older age, due to limitations in both cross-sectional (Schellenberg, 2015; Swaminathan and Schellenberg, 2018; see Hanna-Pladdy and MacKay, 2011) and intervention (Román-Caballero et al., 2018) designs. However, studies with both design types contribute to the overall picture of musical training influencing WM and speech-on-speech perception in older age to some degree.

Though effect sizes vary, cross-sectional evidence has revealed better WM performance in older adult musicians compared to non-musicians (see Table 1). In the studies that directly assess WM, several have reported significantly higher digit span (Mansens et al., 2018; Gray and Gow, 2020; Zhang et al., 2021), visuospatial span (Amer et al., 2013) and better performance on measures of WM maintenance and recall with older musicians (Parbery-Clark et al., 2011). There is evidence to also suggest increased efficiency for older musicians in tasks that indirectly measure WM, or in which WM is an important underpinning of task success. Better problem solving and reasoning (Hanna-Pladdy and Gajewski, 2012; Gray and Gow, 2020), faster and more accurate performance during auditory Stroop and reading with distraction tasks (Amer et al., 2013), superior sustained attention (Tierney et al., 2020), as well as advantages in speech-on-speech (Parbery-Clark et al., 2011; Zendel et al., 2019; Zhang et al., 2021) and verbal learning and fluency (Mansens et al., 2018) have all been reported. Additionally, those studies in which no group differences in digit span were found also revealed a musician advantage in short delayed recall tasks and letter-number sequencing in the same population (Hanna-Pladdy and MacKay, 2011; Hanna-Pladdy and Gajewski, 2012). The combined findings could allude to the limitations in relying on span tasks as a measure of WM, given that it is difficult to parse the temporal components of WM processing, within which older musicians may differ depending on the several factors that account for their musical experience and aptitude. This also highlights the difficulties associated with these studies adopting the commonly used “years of experience” (Hanna-Pladdy and MacKay, 2011; Hanna-Pladdy and Gajewski, 2012; Amer et al., 2013; Gray and Gow, 2020), or similarly weak “yes” or “no” musical training survey items (Mansens et al., 2018) as grouping criteria, as studies cannot then draw within-group comparisons between differing engagement in musical training and those temporal components of WM. Nevertheless, the broad cross-sectional data indicates a positive relationship between musical training and components of WM in older age, and studies that included additional grouping criteria based on the age of onset and current musical activity support the findings (Parbery-Clark et al., 2011; Zhang et al., 2021).

**TABLE 1:** Overview of selected literature organized by theme.

WM and Musical training		Total Sample					Results	
Authors	Year	Size	Age	Sex	Groups	Criteria	Outcome	Findings
Amer et al.	2013	*N* = 42	≥50 years	NA	Musicians (*n* = 18) Non-musicians (*n* = 24)	Years of experience (≥10 years)	-AST -Simon Task -VST -GNG -Reading with Distraction	Musicians outperformed non-musicians on the AST, VST, and reading with distraction. No group differences reported in the Simon Task or GNG
Bugos et al.	2007	*N* = 31	≥60 years	26% Female	Experimental (*n* = 15) Control (*n* = 16)	6-month individualized piano instruction (IPI) program. Participants took part in one 30-min lesson per week and practiced for a minimum of 3 additional hours.	-TMT -DS -TDS -BDI -LNS	Significant improvement for musicians in TMT and DS, as well as better post-test performance than non-musicians. No significant improvement or post-test performance in the TDS, BDI, and LNS
Gray and Gow	2020	*N* = 60	≥60 years	53% Female	Musicians (*n* = 30) Non-musician (*n* = 30)	Years of experience (=10 years)	-CWS -Spatial Reasoning -TMT -Abstract Reasoning -SLCT -TDS	Musicians outperformed non-musicians in measures of CWS, spatial reasoning, abstract reasoning, and SLCT, as well as TDS. No group differences in TMT once scores were adjusted for multiple comparisons.
Hanna-Pladdy and MacKay	2011	*N* = 70	≥60 years	50–60% Female	High-activity musicians (*n* = 22) Low-activity musicians (*n* = 27) Non-musicians (n= 21)	High activity musicians - ≥10 years of experience Low-activity - 5–10 years of experience	-TDS -LNS -WMS -VR -SS -TMT -BNT -L&S fluency -AMNART -CVLT	High-activity musicians performed better than low-activity, and non-musicians in WMS delayed recall, TMT and BNT word retrieval. No group differences in the TDS, LNS, SS, L&S, AMNART and CVLT were reported.
Hanna-Pladdy and Gajewski	2012	*N* = 70	≥59 years	NA	Musicians (*n* = 33) Non-musicians (*n* = 37)	Years of experience (≥10 years)	-LNS -TDS -TMT -L&S fluency -BNT -CVLT-II -WCST -Tower Task -GP	Musicians displayed higher scaled scores in the LNS, which was predicted by earlier age of onset. Musicians also performed better in letter fluency, CVLT short delay and tower task No group differences in the TDS, TMT, BNT and WCST were reported.
Mansens et al.	2018	*N* = 1101	≥55 years	52% Female	Musicians (*n* = 277) Non-musicians (*n* = 824)	Musicians classed as such if they answered ‘Yes’ to the question ‘Do you make music?’ in LASA Physical Activity questionnaire	-TDS -Fluency -AVLT	Musical instrumentalists displayed greater TDS than vocalists and non-musicians and better performance in fluency and AVLT, though no difference reported in recall condition of AVLT.
Seinfeld et al.	2013	*N* = 41	≥60 years	NA	Experimental (*n* = 25) Controls (*n* = 16)	4-month group piano intervention. Included one 90-min lesson and an extra 4-h practice per week. Active controls took part in a variety of leisure activities	-TDS -FT -GP -TMT -SDMT -SCWT	Musical group showed improvements in FT, TMT and SCWT, but only outperformed the control in SCWT. Groups did not differ in the TDS, GP, or SDMT
**SOS and musical training**								
Mussoi	2021	*N* = 31	=65 years	NA	Musicians (*n* = 15) Non-musicians (*n* = 16)	(a) Started musical training before the age of 10 (b) At least 5 years formal training (c) Currently practicing at least 3 h per week	-TDS -QuickSIN -HINT -SPIN-R	Musicians and non-musicians did not differ in the QuickSIN, though low WM capacity participants displayed greater SNR loss. No group differences in the TDS, HINT, SPIN-R
Tierney et al.	2020	*N* = 69	16–65 years	46% Female	NA Correlational design	Years of experience used as primary predictor	-AST -SASA -CRM -BAT	Musicians outperformed non-musicians on SASA and BAT, though BAT reliability score was very low and results to be interpreted with caution. Musicians also performed better on CRM and performance correlating with SASA scores.
Parbery-Clark et al.	2011	*N* = 37	45–65	NA	Musicians (*n* = 18) Non-musicians (*n* = 19)	(a) Started musical training before the age of 9 (b) Currently active at least three times per week	-HINT -QuickSIN -WIN -WJ-III -VWM -BM	Musicians outperformed non-musicians on the HINT, QuickSIN WIN, WJ-III, and BM. HINT and QuickSIN performance correlated with better WJ-III scores. No group differences were reported in the VWM
Zendel et al.	2019	*N* = 34	≥55 years	71% Female	Experimental (*n* = 13) Video Game control (*n* = 8) Control (*n* = 13)	6-month piano training intervention in which participants took part in app-based lessons in their own home. Practice took place for 30 min, 5-days a week.	Participants presented with competing multitalker scenarios and asked to recall target words	Behavioral findings revealed the music group outperformed both controls. EEG findings indicated musical training enhanced the N1 component during passive listening. During active listening, authors related enhanced P300 to improved resource allocation.
Zhang et al.	2021	*N* = 77	≥57	51% Female	Musicians (*n* = 48) Non-Musicians (*n* = 29) (Also analyzed a group of younger adults)	Musicians active in conservatories, choirs, and orchestras	-TDS -AST -SIN	Older instrumentalists displayed significantly greater TDS and better SIN than non-musicians and vocalists. Years of experience associated with better TDS, and this correlated with better SIN in older musicians. No group differences in AST were reported

*AST, Auditory Stroop task; VST, Visuospatial task; GNG, Go/No Go task; TMT, Trail-Making Task; DS, Digit Symbol task; TDS, Total Digit Span; BDI, Beck Depression Inventory; LNS, Letter Number Sequencing; CWS, Color-Word Stroop Task; SLCT, Single Letter Cancellation Task; WMS-VR, Wechsler Memory Scale – Visual Reproduction; BNT, Boston Naming Test; SS, Spatial Span; L&S fluency, Letter fluency and semantic fluency; AMNART, American Adult Reading Test; CVLT, California Verbal Learning Task; Tower, D-KEFS Tower Task; GP, Grooved pegboard task; WCST, Wisconsin Card Sorting Task; AVLT, Auditory Verbal Learning Test; FT, Finger Tapping test; SDMT, Symbol Digit Modalities test; HINT, Hearing in Noise Task; SPIN-R, Revised Speech Reception in Noise task; SASA, Sustained Auditory Selective Attention Task; CRM, Coordinate Response Measure; VWM, Visual Working Memory; BM, Backwards Masking task; BAT, Beat Alignment Test; WJ-III, Woodcock-Johnson III; RST, Reading Span task; SOS, Speech-on-speech perception.*

Musical intervention studies with older adults often report positive results with regards to WM as well. Following 6 weeks of one-to-one piano lessons combined with 3 h of practice per week, Bugos et al. (2007) reported better digit-span performance for musically trained older adults over controls that was maintained at a 6-month follow-up. Elsewhere, older adults who engaged in 6 months of weekly group piano lessons improved within-group digit-span performance whereas controls did not; there was, however, no between-group difference at post-test (Seinfeld et al., 2013). Although promising in terms of improving WM in older age and maintaining improvements once musical training stopped, more intervention studies are needed to determine causality.

## Musical Training and Preserved Alpha-Theta Activity in Older Age?

In older age, benefits in WM related to musical training have been associated with reduced distractor interference and better inhibitory control (Moussard et al., 2016) reputedly originating from the PFC (Alain et al., 2018). As noted, the PFC is thought to underpin alpha recruitment during sensory gating and functional inhibition (Klimesch et al., 2007; Manza et al., 2014; Misselhorn et al., 2019). Beyond this, the relationship between musical training and WM-related alpha-theta activity is still somewhat unclear and to our knowledge, no research has explored musical training-related modulations of alpha recruitment during active inhibition (see Yurgil et al., 2020), especially later in life. However, reported phenomena from younger adults hint that musical training may preserve alpha-theta oscillatory activity. Increased alpha-theta connectivity has been reported in younger adult musicians over non-musicians and is thought to be involved in the propagation of information across long-range brain networks *via* the PFC (Klein et al., 2016). Evidence also suggests a positive effect of musical training on theta-activity specifically related to verbal WM encoding and retrieval. For instance, recent research saw musically trained younger adults outperform non-musicians during a measure of semantic integration and recall of newly learned words, where better performance correlated with increased theta connectivity between ventral and dorsal speech streams (Dittinger et al., 2018). Moreover, increased theta-activity was observed to correlate with the number of years of musical training. Similarly, younger adult musicians performed better than non-musicians in a verbal WM learning and recall task, and increased theta-activity correlated with better performance (Cheung et al., 2017). Next to this oscillation-specific evidence, recent behavioral studies reported positive association between musical training and inhibition (Moreno and Farzan, 2015), as well as musical training benefits in gating information during WM updating (Okada and Slevc, 2018).

The limited literature available from younger adults seems to align with the observed relationship between musical training and WM in older age. Taken together, this supports the idea that musical training may preserve WM-related alpha-theta activity longer into older age. In turn, age-related decline may be delayed from the perspective of the ELU, we suggest that preserved WM-related oscillatory activity in older adults may allow for better explicit repair of mismatched phonological binding during speech-on-speech perception: Preserved alpha-activity may protect from age-related decline in active inhibition of distractor speech during pre-diction. Similarly, we argue that preserved theta-activity in older adults may contribute to more successful post-diction through protection from declines in storage and manipulation of a degraded speech signal, and retrieval of appropriate semantic and lexical representations to understand target speech.

In line with this idea, there is a small body of research reporting improved speech on speech in older adults with musical training compared to adults with no musical training. Improvements in speech-on-speech perception for a group of 69-year-old adults following 6 months of piano lessons were thought to be mediated by benefits in WM (Worschech et al., 2021). Findings that revealed greater post-intervention allocation of attentional resources during speech-on-speech perception for those who underwent musical training, support this view (represented by enhanced attention-related ERP components; Zendel et al., 2019). Better verbal digit-span performance also reputedly mediated better speech-on-speech performance for older musicians in a recent cross-sectional study (Zhang et al., 2021). In Zhang et al. (2021), differences in speech-on-speech were less pronounced in younger musicians, and correlations between WM metrics and improved speech-on-speech perception were more pronounced in older adults compared to younger adults. Given that WM contains a general executive attention component (Chung et al., 2018), WM differences were perhaps reflected in another study when older musicians reportedly outperformed non-musicians during a speech-on-speech perception task, with measures of attentional control accounting for 54% of variance (Tierney et al., 2020). In one recent study where neither musical training nor WM was found to correlate with speech-on-speech (Mussoi, 2021), the author acknowledged that the study was underpowered.

## Limitations

In this mini-review, we do not present comprehensive accounts of speech perception models and make no claims about how the current perspective for the role of WM and related oscillatory activity relates to other models (e.g., Grossberg, 1980; McClelland and Elman, 1986) or neurocognitive frameworks (e.g., Kotz and Schwartze, 2010; Strauß et al., 2014b; Meyer, 2018). We do not fully review all neural mechanisms related to aging (see Cabeza et al., 2018), speech-on-speech processing or hearing loss (e.g., Anderson et al., 2013), nor WM (see Baddeley, 2012; D’Esposito and Postle, 2015; Oberauer, 2019; Xu, 2021). There has been no exhaustive evaluation of WM-related oscillatory activity literature, and the appraisal of the aging, musical training, speech-on-speech, and WM literature is limited to how they intersect. This is in part due to the exploratory nature of this framework, which aims only to provide a direction for future research.

## Summary and Research Perspectives

Our mini-review suggests that within the scope of the ELU model, pre-diction and post-diction components may be underpinned by WM-related alpha and theta activity, respectively. As WM function and associated alpha-theta oscillatory activity can decline during normal aging (Rondina et al., 2019), and lower alpha power is related to a lessened ability to inhibit irrelevant information (ElShafei et al., 2020), we argue that musician benefits to WM in older age may relate to preserved top-down modulation of alpha oscillations in sensory regions when understanding speech-on-speech and enhanced theta-activity relating to WM maintenance and retrieval. Specifically, preserved alpha-band activity could thus improve information gating through active inhibition and increased effort during the ELU’s pre-diction, while theta activity underpins lexical retrieval and the reconstruction of limited perceptual information during the ELU’s post-diction.

Our account highlights the need for further understanding of the relationship between WM, speech-on-speech perception, and musical training in older age. We suggest future musical training paradigms targeting speech-on-speech perception in older adults should not only limit to performance measures of music and speech perception, but also measure WM-related alpha-theta oscillations during speech-on-speech tasks. This will determine whether improvements in WM dictate any observed advantage in older musicians over non-musicians in speech-on-speech perception, and whether improvements in WM dictate this in turn. Additionally, the time course of WM-related alpha-theta oscillatory activity may be able to reflect real-time occurrence of the ELU’s pre-diction and post-diction.

Should the positive relationship between musical training, WM and speech-on-speech perception be causal, this would be extremely promising, not only to clarify inconsistencies in the literature, but also at the societal level. With an increasing life expectancy and aging population, the possible benefits of musical training, whether achievable earlier or later in life, are important to society. Future research addressing online recruitment of WM-related neural resources such as alpha-theta activity during speech-on-speech perception tasks could further help to understand this relationship. Causation can be established by musical intervention studies, and musician/non-musician cross-sectional studies should carefully address heterogeneity in WM capabilities and musical backgrounds of older adults. The knowledge of what effects occur due to age-related sensory changes and what effects due to age-related cognitive changes can be gained with experimental designs that utilize different groups of older individuals with varying hearing status, ranging from age minimal to age typical hearing loss. This new information from such studies can lead to better ways of utilizing successful sensory-cognitive integration during speech-on-speech perception in terms of cognitive compensation. This may also provide an avenue for future customized training and rehabilitation tools for older adults with or without presbycusis.

## Author Contributions

RG, AS, and EH conceived of the research. All authors contributed to the manuscript and contributed to the article and approved the submitted version.

## Conflict of Interest

The authors declare that the research was conducted in the absence of any commercial or financial relationships that could be construed as a potential conflict of interest.

## Publisher’s Note

All claims expressed in this article are solely those of the authors and do not necessarily represent those of their affiliated organizations, or those of the publisher, the editors and the reviewers. Any product that may be evaluated in this article, or claim that may be made by its manufacturer, is not guaranteed or endorsed by the publisher.
