# Supplementary material for: Working-Memory, Alpha-Theta Oscillations and Musical Training in Older Age: Research Perspectives for Speech-on-speech Perception
Source: Front Aging Neurosci. 2022 May 11;14:806439. doi: 10.3389/fnagi.2022.806439 (PMC9131017; doi:10.3389/fnagi.2022.806439)
Supplement: Supplementary file 1 [file Table_1.docx]

Table S1. Overview of selected literature organized by theme

| WM & Musical training |  | Total Sample |  |  |  |  | Results | |
| --- | --- | --- | --- | --- | --- | --- | --- | --- |
| Authors | Year | Size | Age | Sex | Groups | Criteria | Outcome | Findings |
| Amer et al. | 2013 | N = 42 | ≥ 50 years | NA | Musicians (n = 18)  Non-musicians (n = 24) | Years of experience (≥ 10 years) | -AST -Simon Task -VST -GNG -Reading with Distraction | Musicians outperformed non-musicians on the AST, VST, and reading with distraction. No group differences reported in the Simon Task or GNG |
| Bugos et al. | 2007 | N = 31 | ≥ 60 years | 26% Female | Experimental (n = 15)  Control (n = 16) | 6-month individualized piano instruction (IPI) program. Participants took part in one 30-minute lesson per week and practised for a minimum of 3 additional hours. | -TMT -DS -TDS -BDI -LNS | Significant improvement for musicians in TMT and DS, as well as better post-test performance than non-musicians. No significant improvement or post-test performance in the TDS, BDI, and LNS |
| Gray & Gow | 2020 | N = 60 | ≥ 60 years | 53% Female | Musicians (n = 30)  Non-musician (n = 30) | Years of experience (≥10 years) | -CWS -Spatial Reasoning -TMT -Abstract Reasoning - SLCT -TDS | Musicians outperformed non-musicians in measures of CWS, spatial reasoning, abstract reasoning, and SLCT, as well as TDS. No group differences in TMT once scores were adjusted for multiple comparisons. |
| Hanna-Pladdy & Mackay | 2011 | N = 70 | ≥60 years | 50-60% Female | High-activity musicians (n = 22) Low-activity musicians (n = 27) Non-musicians (n= 21) | High activity musicians - ≥10 years of experience  Low-activity - 5-10 years of experience | -TDS -LNS -WMS -VR -SS -TMT -BNT -L&S fluency -AMNART -CVLT | High-activity musicians performed better than low-activity, and non-musicians in WMS delayed recall, TMT and BNT word retrieval. No group differences in the TDS, LNS, SS, L&S, AMNART and CVLT were reported. |
| Hanna-Pladdy & Grajewski | 2012 | N = 70 | ≥59 years | NA | Musicians (n = 33) Non-musicians (n = 37) | Years of experience (≥10 years) | -LNS -TDS -TMT -L&S fluency -BNT -CVLT-II -WCST -Tower Task -GP | Musicians displayed higher scaled scores in the LNS, which was predicted by earlier age of onset. Musicians also performed better in letter fluency, CVLT short delay and tower task No group differences in the TDS, TMT, BNT and WCST were reported. |
| Mansens et al. | 2018 | N = 1101 | ≥ 55 years | 52% Female | Musicians (n = 277) Non-musicians (n = 824) | Musicians classed as such if they answered ‘Yes’ to the question ‘Do you make music?’ in LASA Physical Activity questionnaire | -TDS -Fluency -AVLT | Musical instrumentalists displayed greater TDS than vocalists and non-musicians and better performance in fluency and AVLT, though no difference reported in recall condition of AVLT. |
| Seinfeld et al. | 2013 | N = 41 | ≥60 years | NA | Experimental (n = 25) Controls (n = 16) | 4-month group piano intervention. Included one 90-minute lesson and an extra 4-hour practice per week. Active controls took part in a variety of leisure activities | -TDS -FT -GP -TMT -SDMT -SCWT | Musical group showed improvements in FT, TMT and SCWT, but only outperformed the control in SCWT. Groups did not differ in the TDS, GP, or SDMT |
| SOS & musical training |  |  |  |  |  |  |  |  |
| Mussoi et al. | 2021 | N = 31 | ≥65 years | NA | Musicians (n = 15) Non-musicians (n = 16) | a) Started musical training before the age of 10 b) At least 5 years formal training c) Currently practicing at least 3 hours per week | -TDS -QuickSIN -HINT -SPIN-R | Musicians and non-musicians did not differ in the QuickSIN, though low WM capacity participants displayed greater SNR loss. No group differences in the TDS, HINT, SPIN-R |
| Tierney et al. | 2020 | N = 69 | 16-65 years | 46% Female | NA Correlational design | Years of experience used as the primary predictor | -AST -SASA -CRM -BAT | Musicians outperformed non-musicians on SASA and BAT, though BAT reliability score was very low and results to be interpreted with caution. Musicians also performed better on CRM and performance correlating with SASA scores. |
| Parbery-Clark et al. | 2012 | N = 37 | 45-65 | NA | Musicians (n = 18) Non-musicians (n = 19) | a)Started musical training before the age of 9  b)Currently active at least three times per week | -HINT -QuickSIN -WIN -WJ-III -VWM -BM | Musicians outperformed non-musicians on the HINT, QuickSIN WIN, WJ-III, and BM. HINT and QuickSIN performance correlated with better WJ-III scores. No group differences were reported in the VWM |
| Zendel et al. | 2019 | N = 34 | ≥55 years | 71% Female | Experimental (n = 13) Video Game control (n = 8) Control (n = 13) | 6-month piano training intervention in which participants took part in app-based lessons in their own home.Practice took place for 30 minutes, 5-days a week. | Participants presented with competing multitalker scenarios and asked to recall target words | Behavioural findings revealed the music group outperformed both controls. EEG findings indicated musical training enhanced the N1 component during passive listening. During active listening, authors related enhanced P300 to improved resource allocation. |
| Zhang et al. | 2020 | N = 77 | ≥57 | 51% Female | Musicians (n = 48)  Non-Musicians (n = 29)  (Also analysed a group of younger adults) | Musicians active in conservatories, choirs, and orchestras | -TDS -AST -SIN | Older instrumentalists displayed significantly greater TDS and better SIN than non-musicians and vocalists. Years of experience associated with better TDS, and this correlated with better SIN in older musicians. No group differences in AST were reported |

| Aging and alpha-theta |  |  |  |  |  |  |  |
| --- | --- | --- | --- | --- | --- | --- | --- |
| ElShafei et al. | 2020 | N = 28 | Young adults (20-29 years) Older adults (61-75 years) | 36% Female | Younger Adults (n = 14)  Older Adults (n = 14) | -CAT | Alpha reduction in relevant visual areas was preserved, but in irrelevant areas, alpha increase was reduced in older but not younger adults, resulting in more distractor costs. |
| Rondina et al. | 2019 | N = 32 | Young adults (M = 24.8) Older adults (M = 65.9) | 50% Female | Younger adults (n = 16)  Older adults (n = 16) | Participants took part in a short-delay visuospatial task. | Significantly slower response times for older adults and during retrieval a decrease in alpha-theta activity, particularly over frontal and parietal regions, thought to result in weakened construction and ability to retrieve memory representations. |
| Strunk et al. | 2017 | N = 43 | Young adults (18-35) Older adults (60-80) | 53% Female | Young adults (n = 22)  Older adults (n = 21) | Ppts took part in visual selective attention task with materials presented concurrently. | Both groups displayed greater memory retrieval for attended over unattended items. Older adults however showed evidence of hyperbinding attended and unattended information.Older adults showed reduced alpha activity than younger adults during the correct rejection phase.For younger adults theta activity correlated with context memory accuracy, but this finding did not extend to older adults. |
| Toth et al. | 2014 | N = 36 | Young adults (18-26) Older adults (60-71) | 78% Female | Younger adults (n = 20)  Older adults (n=16) | Visual delayed match to sample task in which ppts were required to memorize colors from a sample array | Older adults’ performance was significantly lower than younger adults and performance decreased with increased memory load. Frontal midline theta activity was significantly reduced in older adults compared to younger adults and this correlated with poorer performance. |
| Aging & SOS |  |  |  |  |  |  |  |
| Anderson et al. | 2013 | N = 120 | ≥55 years | 59% | NA- Correlational design | -AST -W-J III - SIN task | For older adults, the greatest predictors of SIN understanding were working memory, short-term memory, and brainstem processing. |
| Goossens et al. | 2017 | N = 42 | Young adults (20-30) Middle age (50-60) Older adults (70-80) | NA | Younger adults (n = 17)  Middle age (n = 15)  Older adults (n = 10) | -LIST | Normal hearing older adults performed worse than both younger and middle age groups across all conditions, but especially in the most cognitively demanding, informational masking conditions. Authors relate findings to the role of working memory proposed in the ELU model. |
| Schoof & Rosen | 2014 | N = 38 | Young adults (19-29) Older adults (60-72) | NA | Younger adults (n = 19)  Older adults (n = 19) | -SIN task -RST -TEA -LDST -TRT -TOWRE | Older adults performed more poorly than younger adults for babble only SIN. However, older adults performed worse than younger adults in RST, TEA, LDST but not TRT or TOWRE. Working memory was not related to SIN outcome. |
| Vermeire et al. | 2019 | N = 60 | Young adults (19-25) Older adults (60-82) | NA | Younger adults (n = 33)  Older adults (n = 27) | -RST -LIST | Younger adults displayed a significantly greater RST span than older adults and performed significantly better than older adults on LIST. Working memory capacity significantly correlated with LIST performance, and participants’ speech reception thresholds. |

Note: *AST = Auditory Stroop task, VST = Visuospatial task, GNG = Go/No Go task, TMT = Trail-Making Task, DS = Digit Symbol task, TDS = Total Digit Span, BDI = Beck Depression Inventory, LNS = Letter Number Sequencing, CWS = Colour-Word Stroop Task, SLCT = Single Letter Cancellation Task, WMS-VR = Weschler Memory Scale – Visual Reproduction, BNT = Boston Naming Test, SS = Spatial Span, L&S fluency = Letter fluency & semantic fluency, AMNART = American Adult Reading Test, CVLT = California Verbal Learning Task, Tower = D-KEFS Tower Task, GP = Grooved pegboard task, WCST = Wisconsin Card Sorting Task, AVLT = Auditory Verbal Learning Test, FT = Finger Tapping test, SDMT = Symbol Digit Modalities test, HINT = Hearing in Noise Task, SPIN-R = Revised Speech Reception in Noise task, SASA = Sustained Auditory Selective Attention Task, CRM = Coordinate Response Measure, VWM = Visual Working Memory, BM = Backwards Masking task, BAT = Beat Alignment Test, CAT = Competitive Attention Task, WJ-III = Woodcock-Johnson III, LIST = Leuven Intelligibility Sentence Test, RST = Reading Span task, TEA = Test of Everyday Attention, LDST = Letter Digit Substitution Test, TRT = Text Reception Test, TOWRE = Test of Word Reading Efficiency*
